# In vivo – in vitro toxicogenomic comparison of TCDD-elicited gene expression in Hepa1c1c7 mouse hepatoma cells and C57BL/6 hepatic tissue

**DOI:** 10.1186/1471-2164-7-80

**Published:** 2006-04-12

**Authors:** Edward Dere, Darrell R Boverhof, Lyle D Burgoon, Timothy R Zacharewski

**Affiliations:** 1 Department of Biochemistry & Molecular Biology, Michigan State University, East Lansing MI, 48824-1319, USA; 2 National Food Safety & Toxicology Center, Michigan State University, East Lansing MI, 48824-1319, USA; 3 Center for Integrative Toxicology, Michigan State University, East Lansing MI, 48824-1319, USA

## Abstract

**Background:**

*In vitro *systems have inherent limitations in their ability to model whole organism gene responses, which must be identified and appropriately considered when developing predictive biomarkers of *in vivo *toxicity. Systematic comparison of *in vitro *and *in vivo *temporal gene expression profiles were conducted to assess the ability of Hepa1c1c7 mouse hepatoma cells to model hepatic responses in C57BL/6 mice following treatment with 2,3,7,8-tetrachlorodibenzo-*p*-dioxin (TCDD).

**Results:**

Gene expression analysis and functional gene annotation indicate that Hepa1c1c7 cells appropriately modeled the induction of xenobiotic metabolism genes *in vivo*. However, responses associated with cell cycle progression and proliferation were unique to Hepa1c1c7 cells, consistent with the cell cycle arrest effects of TCDD on rapidly dividing cells. In contrast, lipid metabolism and immune responses, representative of whole organism effects *in vivo*, were not replicated in Hepa1c1c7 cells.

**Conclusion:**

These results identified inherent differences in TCDD-mediated gene expression responses between these models and highlighted the limitations of *in vitro *systems in modeling whole organism responses, and additionally identified potential predictive biomarkers of toxicity.

## Background

Advances in microarray and related technologies continue to revolutionize biomedical research and are being incorporated into toxicology and risk assessment. These technologies not only facilitate a more comprehensive elucidation of the mechanisms of toxicity, but also support mechanistically-based quantitative risk assessment [1-5]. In addition, these technologies are being used to develop predictive toxicity screening assays to screen drug candidates with adverse characteristics earlier in the development pipeline in order to prioritize resources and maximize successes in clinical trials [6-8]. Comparable screening strategies are also being proposed to rank and prioritize commercial chemicals, natural products, and environmental contaminants that warrant further toxicological investigation. Traditionally, rodent models or surrogates for ecologically-relevant species are typically used in regulatory testing. However, public and regulatory pressure, especially in Europe, seek to minimize the use of animals in testing [9]. Similar policies in the US, such as the ICCVAM Authorization Act of 2000, provide guidelines to facilitate the regulatory acceptance of alternative testing methods. These initiatives combined with the need to assess an expanding list of drug candidates and commercial chemicals for toxicity, have increased demand for the development and implementation of high-throughput *in vitro *screening assays that are predictive of toxicity in humans and ecologically-relevant species.

Various *in vitro *hepatic models including the isolated perfused liver, precision cut liver slices, isolated primary liver cells and a number of immortalized liver cell lines, have been used as animal alternatives [10]. In addition to providing a renewable model, *in vitro *systems are a cost-effective alternative and are amenable to high-throughput screening. These models, particularly immortalized cell lines, also allow for more in-depth biochemical and molecular investigations, such as over-expression, knock-down, activation or inhibition strategies, thus further elucidating mechanisms of action. However, inherent limitations in the ability of cell cultures to model whole organism responses must also be considered when identifying putative biomarkers for high-throughput toxicity screening assays, and elucidating relevant mechanisms of toxicity that support quantitative risk assessment. Despite several *in vitro *toxicogenomic reports [11-13], few have systematically examined the ability of *in vitro *systems to predict *in vivo *gene expression profiles in response to chemical treatment [10,14].

2,3,7,8-Tetrachlorodibenzo-*p*-dioxin (TCDD) is a widespread environmental contaminant that elicits a number of adverse effects including tumor promotion, teratogenesis, hepatotoxicity, and immunotoxicity as well as the induction of several metabolizing enzymes [15]. Many, if not all of these effects, are due to alterations in gene expression mediated by the aryl hydrocarbon receptor (AhR), a basic-helix-loop-helix-PAS (bHLH-PAS) transcription factor [15,16]. Ligand binding to the cytoplasmic AhR complex triggers the dissociation of interacting proteins and results in the translocation of the ligand-bound AhR to the nucleus where it heterodimerizes with the aryl hydrocarbon receptor nuclear translocator (ARNT), another member of the bHLH-PAS family. The heterodimer then binds specific DNA elements, termed dioxin response elements (DREs), within the regulatory regions of target genes leading to changes in expression that ultimately result in the observed responses [17]. Although the role of AhR is well established, the gene regulatory pathways responsible for toxicity are poorly understood and warrant further investigation to assess the potential risks to humans and ecologically relevant species.

Hepa1c1c7 cells and C57BL/6 mice are well-established models routinely used to examine the mechanisms of action of TCDD and related compounds. In this study, TCDD-elicited temporal gene expression effects were systematically compared in order to assess the ability of Hepa1c1c7 cells to replicate C57BL/6 hepatic tissue responses. Our results indicate that several phase I and II metabolizing enzyme responses are aptly reproduced. However, many responses were model-specific and reflect inherent *in vitro *and *in vivo *differences that must be considered in mechanistic studies and during the selection of biomarkers for developing toxicity screening assays.

## Results

### In vitro microarray data analysis

**Figure 1:**
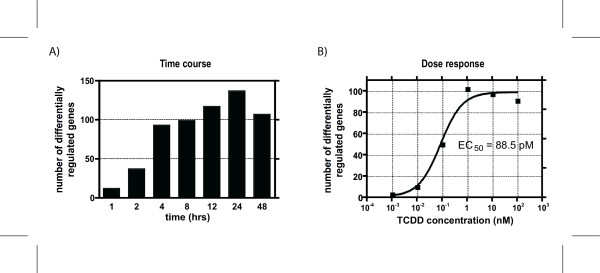
Number of genes differentially regulated (P 1(*t*) > 0.9999 and Ifold changel > 1.5-fold) as measured by microarray analysis for the (A) time course and (B) and dose-response studies in mouse hepatoma Hepa1c1c7 cells. For the time course study, cells were treated with 10 nM TCDD and harvested at 1, 2, 4, 8, 12, 24 or 48 hrs after treatment. Cells for the 12 hr dose-response study were treated with 0.001, 0.01, 0.1, 1.0, 10 and 100 nM of TCDD

Temporal gene expression profiles were assessed in Hepa1c1c7 wild type cells following treatment with 10 nM TCDD using cDNA microarrays with 13,362 spotted features. Empirical Bayes analysis of the *in vitro *time course data identified 331 features representing 285 unique genes with a P1(*t*) value greater than 0.9999 at one or more time points, and differential expression greater than ± 1.5 fold relative to time-matched vehicle controls. The number of differentially regulated genes gradually increased from 1 to 24 hrs, followed by a slight decrease at 48 hrs (Figure 1A). *In vitro *dose-response data performed at 12 hrs with TCDD covering 6 different concentrations (0.001, 0.01, 0.1, 1.0, 10 and 100 nM), identified 181 features representing 155 unique genes (P1(*t*) > 0.9999 and an absolute fold change > 1.5 at one or more doses; Figure 1B). Complete *in vitro *time course and dose-response data are available in Additional file 1 and 2, respectively.

As a control, the gene expression effects elicited by 10 nM TCDD in ARNT-deficient c4 Hepa1c1c7 mutants [18] were examined at 1 and 24 hrs (data not shown). Only ATPase, H^+ ^transporting, V1 subunit E-like 2 isoform 2 (Atp6v1e2) and SUMO/sentrin specific peptidase 6 (Senp6) exhibited a significant change in expression using the same criteria (P1(*t*) > 0.9999 and an absolute fold change > 1.5). Neither Atp6v1e2 nor Senp6 were among the active genes in wild-type Hepa1c1c7 cells or in C57BL/6 liver samples [19]. These results provide further evidence that the AhR/ARNT signaling pathway mediates TCDD-elicited gene expression responses, which are consistent with *in vivo *microarray results with AhR knockout mice [20].

**Figure 2:**
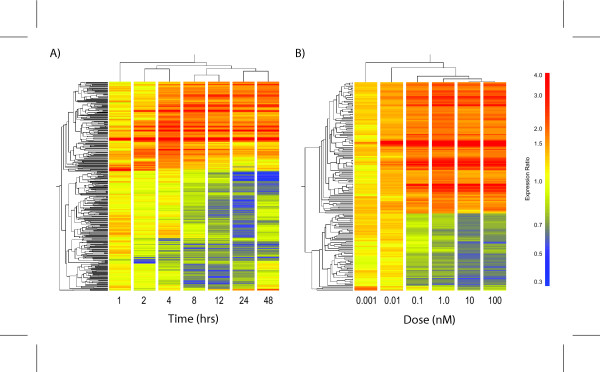
Hierarchical clustering of the differentially regulated gene lists for A) temporal and B) dose-response microarray studies in mouse hepatoma Hepa1c1c7 cells. The results illustrate time and dose-dependent clustering patterns. From the A) temporal results, the early (2 hr and 4 hr), intermediate (8 hr and 12 hr) and late (24 hr and 48 hr) time points cluster separately while the 1 hr time point clusters alone. Results from the B) dose-response show that highest doses clustered together, while the remaining doses branched out in a dose-dependent manner

Hierarchical clustering of the genes expressed in Hepa1c1c7 time course indicate that 2 and 4 hrs were most similar, as were 8 and 12 hrs, and 24 and 48 hrs, while the 1 hr time point was segregated (Figure 2A). A strong dose-response relationship was also evident with clusters sequentially branching out with increasing concentration (Figure 2B). At 12 hrs, 117 genes were differentially expressed with 112 exhibiting a dose-dependent response. Moreover, the fold changes measured in both the time course and dose-response studies using 10 nM TCDD were comparable. For example, xanthine dehydrogenase (Xdh) and NAD(P)H dehydrogenase, quinone 1 (Nqo1) were induced 2.39- and 4.89-fold respectively in the time course and 2.93- and 4.71-fold in the dose-response study. There is a strong correlation (R = 0.97) between the differentially expressed genes at 12 hrs in the time course with the differentially regulated genes in the dose-response study at 10 nM, demonstrating the reproducibility between independent studies and providing further evidence that these genes are regulated by TCDD.

**Figure 3:**
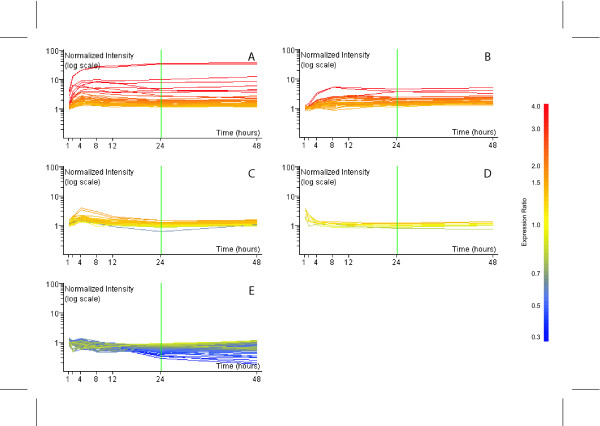
K-means clustering of temporally differentially regulated genes *in vitro*. Five k-mean clusters corresponding to (A) up-early and sustained, (B) up-intermediate and sustained, (C) up-regulated intermediate, (D) up-regulated immediate and (E) down-regulated late. Time and expression ratio are indicated on the *x*- and y-axis respectively. The color of individual gene expression profiles reflects the expression ratio observed at 24 hrs

The list of temporally regulated genes was subjected to *k*-means clustering using the standard correlation distance metrics. Five *k*-means clusters best characterized the dataset and identified clusters representing A) up-regulated early and sustained, B) up-regulated intermediate and sustained, C) up-regulated intermediate, D) up-regulated immediate and E) down-regulated late (Figure 3). These were comparable to the *k*-means clusters identified in hepatic tissue of C57BL/6 mice following treatment with 30 μg/kg TCDD [19]. Although, no discernable functional category is over-represented in any one cluster, the sustained up-regulation of early (Cluster A) and intermediate (Cluster B) responding genes include classic TCDD-responsive genes such as cytochrome P450, family 1, subfamily a, polypeptide 1 (Cyp1a1), Xdh and Nqo1. Many down-regulated late genes were associated with cell cycle regulation such as myelocytomatosis oncogene (Myc). Additionally, targets of Myc, including cyclin D1 and ornithine decarboxylase (Odc1), were also down-regulated suggesting a mechanism for cell cycle arrest [21-23], a common *in vitro *response to TCDD.

### Classification of gene expression responses for common regulated genes

Using the same filtering criteria (P1(*t*) > 0.9999 and an absolute fold change > 1.5), 678 features representing 619 unique genes were differentially expressed as previously reported in a time course study conducted in hepatic tissue from C57BL/6 mice orally gavaged with 30 μg/kg TCDD [19]. The number of responsive *in vivo *genes and their temporal expression patterns closely paralleled the results from this *in vitro *study. The fewest number of active genes was observed at 2 hrs, followed by a large increase at 4 hrs, which was sustained to 72 hrs. However, the substantial increase in expressed *in vivo *genes at 168 hrs was attributed to triglyceride accumulation and immune cell infiltration, which was not observed in Hepa1c1c7 cells. This list of 619 of *in vivo *genes served as the basis for subsequent comparisons against TCDD-elicited *in vitro *responses.

**Figure 4:**
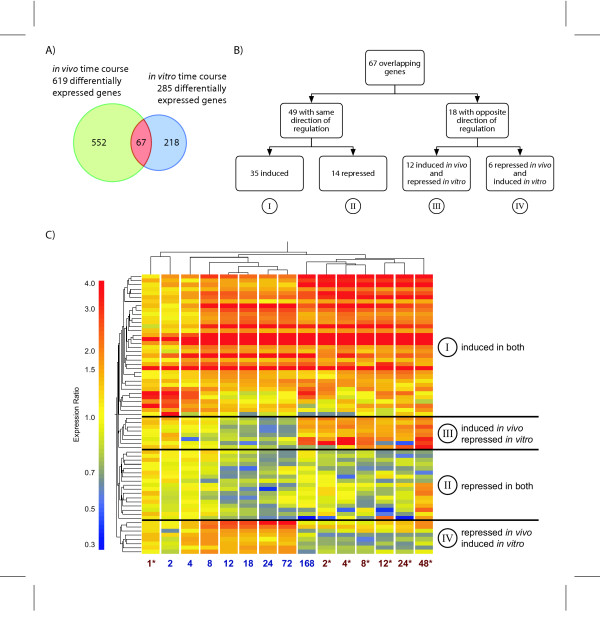
Comparison of common significant *in vitro *and *in vivo *TCDD elicited time-dependent gene expression changes. A) 285 differentially regulated *in vitro *genes and 619 differentially regulated *in vivo *genes were identified, with 67 genes common to both studies. B) The temporal gene expression profiles from both studies were categorized into (I) induced in both, (II) repressed in both, (III) induced *in vivo *and repressed *in vitro*, and (IV) repressed *in vitro *and induced *in vivo*. C) Hierarchical clustering identified similar classification groups. Clustering across both time and model, separated samples from *in vitro *and *in vivo*, with the exception of the early time points from both studies (1 hr *in vitro *and 2 hr *in vivo*), which clustered together. * identifies *in vitro *time points

Comparison of *in vitro *and *in vivo *differentially expressed gene lists identified common and model specific responses (Figure 4A). TCDD treatment resulted in a total of 838 regulated genes in either model and with 67 common to both. TCDD elicited 218 gene expression changes unique to Hepa1c1c7 cells while 552 genes were specific to C57BL/6 hepatic samples. Although 67 genes were regulated in both models, not all possessed similar temporal patterns of expression. Contingency analysis using a 2 × 2 table and the χ^2 ^test resulted in a p-value < 0.001 (α = 0.05) illustrate a statistically significant association between the lists of differentially regulated genes *in vitro *and *in vivo*. Further stratification revealed genes that were either induced in both models (class I), repressed in both models (class II), induced *in vivo *while repressed *in vitro *(class III), or repressed *in vivo *while induced *in vitro *(class IV; Figure 4B). Genes regulated in a similar fashion in both models (classes I and II) accounted for 49 of the 67 common active genes, while the remaining genes exhibited divergent expression profiles (classes III and IV). Hierarchical clustering of the temporal expression values for the 67 overlapping genes identified the same four classes (Figure 4C). The pattern across model and time illustrates that the earliest time points (i.e. 1 hr *in vitro *and 2 hr *in vivo *time points) cluster together while the remaining clusters branch into *in vitro *or *in vivo *clusters according to time. These results suggest that potential biomarkers of acute TCDD-mediated responses may best be predicted by the immediate-early *in vitro *gene responses.

*In vitro *and *in vivo *induced genes (class I) include xenobiotic and oxidoreductase enzymes such as abhydrolase domain containing 6 (Abhd6), Cyp1a1, dehydrogenase/reductase (SDR family) member 3 (Dhrs3), Nqo1, prostaglandin-endoperoxide synthase 1 (Ptgs1), UDP-glucose dehydrogenase (Ugdh) and Xdh (Table 1). These genes have previously been reported to be TCDD-responsive [19,24], with Cyp1a1 and Nqo1 being members of the "AhR gene battery" [25]. Glutathione S-transferase, alpha 4 (Gsta4) was also induced *in vitro *and *in vivo*, 1.7- and 2.0-fold respectively, consistent with TCDD-mediated induction of phase I and II metabolizing enzymes. Of the 35 genes responding similarly in both models, approximately 71% of were similarly up-regulated (class I) while the remaining genes were repressed across both models (class II). Repressed class II genes include minichromosome maintenance deficient 6 (Mcm6), glycerol kinase (Gyk) and ficolin A (Fcna) (repressed 1.6-, 1.6- and 1.7-fold *in vitro*, respectively). Overall, repressed genes did not share any common discernable biological function.

**Table 1:** Classification of common differentially regulated temporal gene expression responses to TCDD in both *in vitro *and *in vivo *models

Accession	Gene name	Gene symbol	Entrez Gene ID	*In vivo*			*In vitro*		
					
				Fold change^a^	Time points^b^	EC50^c,d ^(μg/kg)	Fold change^a^	Time points^b^	EC50^c,d ^(pM)
I) Induced both *in vivo *and *in vitro*^e^									
BE689910	RIKEN cDNA 2310001H12 gene	2310001H12Rik	69504	2.7	2^f^, 168	48.02	3.9	1^f^	ND
BF226070	RIKEN cDNA 2600005C20 gene	2600005C20Rik	72462	2.1	4, 12, 18, 24^f^, 72, 168	2.18	2.3	4, 8, 12^f^, 24, 48	265.50
AI043124	RIKEN cDNA 2810003C17 gene	2810003C17Rik	108897	1.6	12^f^	37.02	1.7	4^f^	ND
AW537038	expressed sequence AA959742	AA959742	98238	7.2	4, 8, 12^f^, 18, 24, 72, 168	1.71	5.2	4, 8^f^, 12, 24, 48	67.79
W34507	abhydrolase domain containing 6	Abhd6	66082	1.7	4, 8^f^, 12, 18, 24, 72, 168	154.30	1.5	48^f^	138.50
NM_026410	cell division cycle associated 5	Cdca5	67849	8.8	4, 8, 12, 18, 24, 72^f^, 168	ND	1.7	4^f^	ND
BG063743	craniofacial development protein 1	Cfdp1	23837	3.6	4, 8, 12^f^, 18, 24, 72, 168	14.27	2.3	4^f^, 8, 12, 24, 48	42.64
AA073604	procollagen, type I, alpha 1	Col1a1	12842	1.7	18, 24, 72^f^	0.65	1.6	4, 8, 12^f^	17.25
NM_009992	cytochrome P450, family 1, subfamily a, polypeptide 1	Cyp1a1	13076	38.4	2, 4, 8, 12, 18, 24^f^, 72, 168	0.05	37.7	1, 2, 4, 8, 12, 24, 48^f^	14.06
BE457542	Dehydrogenase/reductase (SDR family) member 3	Dhrs3	20148	2.0	4, 8, 12^f^, 18, 72, 168	0.67	1.5	8^f^	2.43
AW552715	DnaJ (Hsp40) homolog, subfamily B, member 11	Dnajb11	67838	1.7	12, 18, 24^f^,168	3.95	1.6	8, 12^f^	9.85
AK015223	dermatan sulphate proteoglycan 3	Dspg3	13516	6.2	4, 8, 12, 18, 24^f^, 72, 168	0.13	8.4	2, 4, 8, 12, 24, 48^f^	16.34
NM_008655	growth arrest and DNA-damage-inducible 45 beta	Gadd45b	17873	4.6	2^f^, 4, 72	133.30	3.7	1^f^, 2	1440.00
W54349	glutathione S-transferase, alpha 4	Gsta4	14860	2.0	18, 24, 72^f^	0.48	1.7	8^f^, 12	56.38
BG067127	interferon regulatory factor 1	Irf1	16362	1.5	168^f^	ND	1.7	2^f^, 4	ND
AA015278	integrin beta 1 (fibronectin receptor beta)	Itgb1	16412	1.6	4, 18, 24, 168^f^	97.23	4.2	4, 8, 12, 24, 48^f^	72.92
AA041752	Jun proto-oncogene related gene d1	Jund1	16478	2.0	12^f^, 18, 24	0.99	2.1	4, 8, 12, 24, 48^f^	50.34
BF538945	lectin, mannose-binding, 1	Lman1	70361	1.9	12, 72, 168^f^	13.49	2.0	4^f^, 8, 24, 48	40.72
BG066626	lipin 2	Lpin2	64898	3.0	4, 12, 24^f^, 72	3.13	2.3	2, 4^f^, 8, 12, 24, 48	23.83
BI440950	leucine rich repeat containing 39	Lrrc39	109245	2.9	2^f^, 4	49.71	3.1	1^f^, 2	68.57
AW413953	mitochondrial ribosomal protein L37	Mrpl37	56280	8.3	2, 4, 8^f^, 12, 18, 24, 72, 168	8.77	2.7	2, 4^f^, 8, 12, 24, 48	49.59
BE623489	NAD(P)H dehydrogenase, quinone 1	Nqo1	18104	4.6	4, 8, 12^f^, 18, 24, 72, 168	1.00	5.2	4, 8^f^, 12, 24, 48	33.74
NM_026550	PAK1 interacting protein 1	Pak1ip1	68083	3.8	4, 8, 12, 18, 24, 72^f^, 168	0.26	2.2	4^f^, 8, 12, 24, 48	7.00
AA152754	prostaglandin-endoperoxide synthase 1	Ptgs1	19224	1.6	168^f^	1.11	2.3	4, 8^f^, 12, 24, 48	37.96
BG063583	solute carrier family 20, member 1	Slc20a1	20515	2.2	2, 4^f^, 8	ND	1.8	2^f^, 4	ND
AJ223958	solute carrier family 27 (fatty acid transporter), member 2	Slc27a2	26458	1.9	12^f^, 18, 24, 72, 168	2.88	2.1	8, 12^f^, 24, 48	17.42
BG066820	solute carrier family 6 (neurotransmitter transporter, taurine), member 6	Slc6a6	21366	1.8	4^f^, 12	2.48	1.7	48^f^	3.06
AI592773	suppression of tumorigenicity 5	St5	76954	1.6	8, 12^f^	28.85	1.7	4^f^, 8, 12	14.69
BG067168	TCDD-inducible poly(ADP-ribose) Polymerase	Tiparp	99929	10.3	2, 4^f^, 12, 18, 24, 72, 168	36.49	6.4	1, 2^f^, 4, 8, 12, 24, 48	18.03
BG065761	tumor necrosis factor, alpha-induced protein 2	Tnfaip2	21928	5.5	2, 4^f^, 12, 18, 72	36.41	6.3	2, 4^f^, 8, 12, 24, 48	41.15
AA067191	UDP-glucose dehydrogenase	Ugdh	22235	3.1	4, 8, 12 ^f^, 18, 24, 72, 168	0.79	1.5	2, 4, 8, 12^f^, 48	4.33
NM_011709	whey acidic protein	Wap	22373	5.9	2, 4, 8, 12, 18, 24, 72, 168^f^	0.12	4.2	2, 4, 8, 12, 24, 48^f^	17.44
BG075778	Xanthine dehydrogenase	Xdh	22436	2.7	4, 8, 12^f^, 18, 24, 72, 168	1.24	2.6	4, 8, 12, 24, 48^f^	34.92
BG073881	zinc finger protein 36, C3H type-like 1	Zfp36l1	12192	2.2	2^f^	ND	1.7	1, 2^f^	2427.00
AA031146	zinc finger protein 672	Zfp672	319475	1.6	4^f^	3.09	1.5	2^f^	ND
									
II) Repressed both *in vivo *and *in vitro*^e^									
BG146493	RIKEN cDNA 6330406L22 gene	6330406L22Rik	70719	-1.5	18^f^	0.51	-1.8	8, 12^f^	25.67
AA140059	DNA methyltransferase (cytosine-5) 1	Dnmt1	13433	-1.9	168^f^	ND	-1.6	8^f^, 12	ND
AI327022	ficolin A	Fcna	14133	-1.6	18, 24^f^	ND	-1.7	12, 24^f^	ND
AA288963	fibrinogen-like protein 1	Fgl1	234199	-1.9	24^f^	ND	-1.5	24^f^, 48	ND
BE626913	GTP binding protein 6 (putative)	Gtpbp6	107999	-3.4	24, 72^f^	ND	-1.7	24, 48^f^	116.40
AA275564	glycerol kinase	Gyk	14933	-1.5	12^f^	10.2	-1.6	24^f^	ND
BG070106	lipocalin 2	Lcn2	16819	-2.8	24^f^	ND	-1.5	24, 48^f^	ND
AW049427	leucine zipper domain protein	Lzf	66049	-1.6	24^f^	ND	-1.6	48^f^	78.29
AA016759	minichromosome maintenance deficient 6	Mcm6	17219	-1.6	18^f^	3.34	-1.6	8^f^	58.04
BF011268	mitochondrial methionyl-tRNA formyltransferase	Mtfmt	69606	-1.8	24, 72^f^, 168	ND	-1.6	24, 48^f^	ND
AA683699	RNA (guanine-7-) methyltransferase	Rnmt	67897	-2.0	12^f^	ND	-1.6	8^f^	ND
	syntrophin, gamma 1	Sntg1	71096	-1.6	24^f^	15.27	-1.7	4^f^	66.61
AA199550	syntaxin 12	Stx12	100226	-1.5	18^f^	ND	-1.6	48^f^	ND
AA047942	thymidine kinase 1	Tk1	21877	-1.7	18^f^, 24, 72	0.34	-2.0	8, 12^f^	153.90
									
III) Induced *in vivo *and repressed *in vitro*^e^									
AA122925	carbonic anhydrase 2	Car2	12349	2.4	12, 72, 168^f^	2.00	-1.8	24^f^, 48	55.96
AI327078	coactosin-like 1	Cotl1	72042	1.6	168^f^	ND	-1.7	24, 48^f^	25.19
NM_007935	enhancer of polycomb homolog 1	Epc1	13831	1.6	168^f^	1.16	-2.7	12, 24^f^	75.21
BC002008	fatty acid binding protein 5, epidermal	Fabp5	16592	3.9	8, 12^f^	2.43	-1.9	8, 12^f^, 24	54.14
NM_026320	growth arrest and DNA-damage- inducible, gamma interacting protein 1	Gadd45gip1	102060	1.8	168^f^	4.67	-1.5	8^f^	40.49
W11419	inhibitor of DNA binding 3	Id3	15903	1.8	168^f^	0.34	-1.5	24, 48^f^	88.83
AA009268	myelocytomatosis oncogene	Myc	17869	3.7	4, 12^f^, 168	5.59	-2.2	2^f^	148.40
NM_011033	poly A binding protein, cytoplasmic 2	Pabpc2	18459	7.0	2^f^	ND	-1.6	12^f^	ND
	REST corepressor 1	Rcor1	217864	1.9	4, 8, 18, 72, 168	3.70	-1.6	24^f^	116.50
BE980584	secretory granule neuroendocrine protein 1, 7B2 protein	Sgne1	20394	3.3	168^f^	0.74	-1.5	48^f^	175.00
AA462951	transcription factor 4	Tcf4	21413	1.6	12^f^, 168	5.77	-1.5	24^f^	74.44
AA003942	tenascin C	Tnc	21923	1.6	168^f^	0.37	-1.8	24^f^, 48	59.34
									
IV) Repressed *in vivo *and induced *in vitro*^e^									
W36712	B-cell translocation gene 2, anti- proliferative	Btg2	12227	-1.8	18^f^, 24	ND	1.5	4^f^	ND
AA174215	cathepsin L	Ctsl	13039	-1.6	24^f^, 72, 168	ND	1.6	8, 48^f^	ND
AA419858	cysteine rich protein 61	Cyr61	16007	-1.6	2^f^	0.07	1.6	8, 48^f^	ND
AW488956	polo-like kinase 3	Plk3	12795	-1.6	4^f^	ND	1.6	4, 48^f^	ND
BG068288	solute carrier organic anion transporter family, member 1b2	Slco1b2	28253	-1.7	8^f^, 12, 18, 24, 72, 168	ND	1.6	4^f^	1.18
NM_011470	small proline-rich protein 2D	Sprr2d	20758	-1.6	18^f^, 72	1.97	1.6	4^f^	ND

^a^Maximum absolute fold change determined by microarray analysis

^b^Time point where genes are differentially regulated with P1(t) > 0.9999 and |fold change| > 1.5

^c^EC50 valued determined from microarray results

^d^ND = not determined from microarray results

^e^Classification groups as defined in Figure 4B

^f^Time point representing the maximum |fold change|

Forty-two of the 67 common differentially expressed genes were dose responsive at 12 and 24 hrs *in vitro *and *in vivo*, respectively, further suggesting the role of the AhR in mediating these responses. Microarray-based EC_50 _values spanned at least 3 orders of magnitude ranging from 0.05 μg/kg to >150 μg/kg *in vivo*, and 0.00118 nM to 2.4 nM *in vitro *(Table 1). Cyp1a1, the prototypical marker of TCDD exposure, had EC_50 _values of 0.05 μg/kg and 0.014 nM, *in vivo *and *in vitro *respectively, and was induced 38-fold in both time course studies. Complete data sets for the *in vivo *time course and dose-responses experiments are available in Additional file 3 and 4.

Of the 67 overlapping genes, 18 exhibited divergent temporal profiles (classes III and IV). Class III contains 12 genes induced *in vivo *but repressed *in vitro*, while 6 were repressed *in vivo *and induced *in vitro *(class IV). Example genes include Myc (class III) and B-cell translocation gene 2 (Btg2, class IV) which are both involved in regulating cell cycle progression [23,26-30]. Myc was induced 3.7-fold *in vivo *and repressed 2.2-fold *in vitro*, while Btg2 was repressed 1.8-fold *in vivo *and induced 1.5-fold *in vitro*.

In addition to the regulated genes common to both models, 218 *in vitro*- and 559 *in vivo*-specific genes were identified. Many of the unique *in vitro *responses are involved in cell cycle regulation, including cyclins D1 and B2 (Table 2). Cyclin D1, which complexes with cyclin-dependent kinase 4 (Cdk4) to regulate the progression from G_1 _to S phase [31,32], was down-regulated early and repressed 1.7-fold to 48 hrs. Furthermore, cyclin B2 and cell division cycle 2 homolog A (Cdc2a) which interact to form an active kinase required for G_2 _promotion, were down-regulated, 1.8-fold and 1.5-fold, respectively. In addition to cell cycle related genes, UDP glucuronosyltransferase 1 family, polypeptide A2 (Ugt1a2), a phase II metabolizing enzyme, was induced 2.8-fold *in vitro*, but not significantly regulated *in vivo*.

**Table 2:** Examples of TCDD-elicited gene expression responses unique to Hepa1c1c7 cells

Accession	Gene name	Gene Symbol	Entrez Gene ID	Fold change^a^	Time points^b ^(hrs)
AA111722	cyclin D1	Ccnd1	12443	-1.7	4, 8, 12, 24^c^, 48
AA914666	cyclin-dependent kinase inhibitor 2B (p15, inhibits CDK4)	Cdkn2b	12579	2.4	4^c^, 8, 48
BC008247	cyclin B2	Ccnb2	12442	-1.8	24^c^
BG064846	cell division cycle 2 homolog A (S. pombe)	Cdc2a	12534	-1.5	12, 24^c^
AA011839	minichromosome maintenance deficient 2 mitotin (S. cerevisiae)	Mcm2	17216	-1.8	8^c^, 12
BG074721	minichromosome maintenance deficient 7 (S. cerevisiae)	Mcm7	17220	-1.7	8, 12^c^, 24
AA003042	myeloblastosis oncogene-like 2	Mybl2	17865	-2.2	8^c^, 12, 24
L27122	UDP glucuronosyltransferase 1 family, polypeptide A2	Ugt1a2	22236	2.8	4, 8, 12^c^, 24, 48

^a^Maximum absolute fold change determined by microarray analysis

^b^Differentially regulated genes with P1(t) > 0.9999 and |fold change| > 1.5

^c^Time point representing the maximum |fold change|

Analysis of the C57BL/6 hepatic time course identified 552 unique genes that were solely regulated *in vivo*. This included TCDD induced transcripts for microsomal epoxide hydrolase 1 (Ephx1) and carbonyl reductase 3 (Cbr3) which both function as xenobiotic metabolizing enzymes. Notch gene homolog 1 (Notch1) and growth arrest specific 1 (Gas1) which are both associated with development and differentiation but serve undetermined roles in the liver, were also induced by TCDD (Table 3). Genes related to immune cell accumulation were also specific to the *in vivo *study, coincident with immune cell accumulation at 168 hr as determined by histopathological examination [19].

**Table 3:** Examples of TCDD-elicited gene expression responses unique to C57BL/6 hepatic tissue

Accession	Gene name	Gene Symbol	Entrez Gene ID	Fold change^a^	Time points^b ^(hrs)
AA170585	carbonic anhydrase 3	Car3	12350	-3.5	12^c^, 18, 24, 168
AK003232	carbonyl reductase 3	Cbr3	109857	2.2	12, 18^c^
AA571998	CD3 antigen, delta polypeptide	Cd3d	12500	-2.4	12, 18^c^, 24, 72, 168
BG072496	ELOVL family member 5, elongation of long chain fatty acids	Elovl5	68801	2.0	8, 12^c^, 18, 24, 72, 168
BG072453	epoxide hydrolase 1, microsomal	Ephx1	13222	1.9	8, 12 18, 24^c^
W84211	growth arrest specific 1	Gas1	14451	-1.9	4, 8, 18, 24, 72, 168^c^
W41175	glycerol phosphate dehydrogenase 2, mitochondrial	Gpd2	14571	-2.3	8, 12, 18, 24, 72^c^, 168
W29265	glutathione S-transferase, alpha 2 (Yc2)	Gsta2	14858	7.2	12, 18, 24, 72^c^, 168
AA145865	lymphocyte antigen 6 complex, locus A	Ly6a	110454	2.5	72, 168^c^
W98998	Notch gene homolog 1 (Drosophila)	Notch1	18128	3.3	2, 4^c^, 8, 12, 18, 24, 72, 168

^a^Maximum absolute fold change determined by microarray analysis

^b^Differentially regulated genes with P1(t) > 0.9999 and |fold change| > 1.5

^c^Time point representing the maximum |fold change|

### Comparison of basal gene expression levels in Hepa1c1c7 cells and hepatic tissue

**Figure 5:**
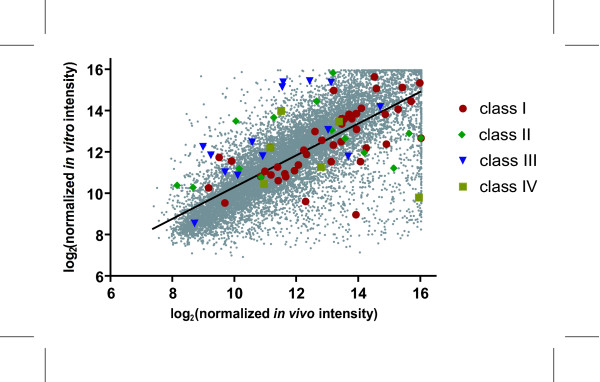
Comparison of Hepalclc7 cell and C57BL/6 hepatic tissue basal gene expression. Untreated samples from Hepalclc7 cells and hepatic tissue from immature ovariectomized C57BL/6 mice taken at 0 hrs were competitively hybridized to the 13,362 feature cDNA microarray. Log2 normalized signal intensities were plotted for *in vitro *versus *in vivo *data to generate the correlation coefficient. The linear correlation coefficient R was 0.75 between *in vitro *and *in vivo *models

In order to further investigate differences in gene expression levels, Hepa1c1c7 cells and C57BL/6 liver samples were directly compared by competitive hybridization on the same array, to identify basal gene expression level differences. Subsequent linear regression analysis of the mean normalized signal intensities from the untreated samples resulted in a correlation value of R = 0.75 (Figure 5), which is consistent with basal gene expression comparisons of various *in vitro *rat hepatic systems against whole livers, where correlation values decreased between liver slices (R = 0.97), primary cells (R = 0.85), BRL3A (R = 0.3) and NRL clone 9 (R = 0.32) rat liver cell lines [10]. Overall, the correlation illustrates reasonable concordance in basal gene expression levels between the two models. However, data points which deviate from the fitted line indicate differences in the basal expression of individual genes between the Hepa1c1c7 cells and hepatic tissue from C57BL/6 mice. Although there are differences, they may be negligible if the TCDD-elicited responses are conserved *in vitro *and *in vivo*. Complete microarray data for the untreated comparisons are available in Additional file 5.

The relative basal expression of the 67 common active features was further investigated (Figure 5). In general, class I (i.e. induced in both models) genes fell close to the regression line, indicating that the basal expression of induced genes were comparable as were their *in vitro *and *in vivo *responses to TCDD. In contrast, basal expression levels of class III genes (i.e. induced *in vivo *while repressed *in vitro*) were generally higher in the Hepa1c1c7 cells, while levels in class II and IV (i.e. repressed in both models and repressed *in vivo *while induced *in vitro*, respectively) genes were scattered around the fitted linear line in Figure 5.

### Quantitative real-time PCR verification of microarray responses

**Figure 6:**
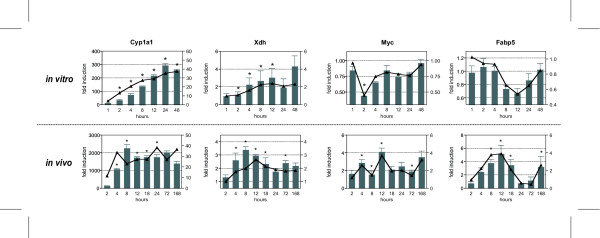
Quantitative real-time PCR verification of *in vitro *and *in vivo *microarray results. The same RNA used for cDNA microarray analysis was examined by QRTPCR. All fold changes were calculated relative to time-matched vehicle controls. Bars (left axis) and line (right axis) represent data obtained by QRTPCR and cDNA microarrays, respectively. Genes are indicated by official gene symbols, and results are the average of four biological replicates. Classes refer to the respective classification categories as illustrated in Figure 4B. Error bars represent the standard error of measurement for the average fold change. *p < 0.05 for QRTPCR

In total, 14 *in vitro *and 24 *in vivo *responsive genes representing common and model-specific genes were verified by quantitative real-time PCR (QRTPCR) (see Additional file 6). Of the selected genes regulated in both models, all displayed temporal patterns comparable to the microarray data (Figure 6). For example, Xdh, Myc and fatty acid binding protein (Fabp5) exhibited good agreement in fold change and temporal expression pattern when comparing microarray and QRTPCR data. However, significant data compression was evident when comparing *in vitro *and *in vivo *Cyp1a1 induction by QRTPCR, although *in vitro *and *in vivo *microarray induction levels were comparable. Previous studies suggest this is likely due to the limited fluorescence intensity range (0 – 65,535) of microarrays resulting in signal saturation and compression of the true magnitude of induction of transcript levels [33,34]. Cross hybridization of homologous probes to a given target sequence on the microarray may also be a contributing factor, especially in comparison to other, more gene-specific measurement techniques [35].

## Discussion

Microarrays have become an invaluable tool in toxicogenomics for comprehensively characterizing gene expression responses following treatment with an environmental contaminant, commercial chemical, natural product or drug as well as for investigating complex mixtures relevant to human and wildlife exposures. An emerging consensus suggests that toxicogenomics will accelerate drug development and significantly improve quantitative risk assessments [36,37]. In addition, toxicogenomics supports the development and refinement of predictive *in vitro *high-throughput toxicity screening assays that can be used as alternatives to traditional *in vivo *testing. Ideally, *in vitro *high-throughput toxicity screens can be used to rank and prioritize drug candidates, environmental contaminants, and commercial chemicals, which warrant further development or testing. Although *in vitro *responses are assumed to reflect a subset of comparable *in vivo *responses, few studies have completed a comprehensive and systematic comparison. This study closely examined two well-established models, and comprehensively compared the TCDD-elicited gene expression to assess the predictive value of *in vitro *systems.

Comparative analysis of Hepa1c1c7 cell and hepatic C57BL/6 microarray data identified 67 differentially expressed genes co-regulated by TCDD. Four classes based on their temporal expression patterns were identified (Figure 4B and 4C), with 42 of the 67 common regulated genes exhibiting dose-response characteristics in both models. *In vitro *EC_50 _values ranged from 0.001182 nM to 2.4 nM, while *in vivo *the values ranged from 0.05 μg/kg to >150 μg/kg. The wide range of EC_50 _values illustrate the varying sensitivity of regulated genes to TCDD in both models.

Hepa1c1c7 cells and hepatic tissue from C57BL/6 mice are the prototypical models used to investigate the mechanisms of action of TCDD and other related compounds and both exhibited the classic induction of phase I and II metabolizing enzymes including Cyp1a1 and Nqo1 [38,39]. Gsta4 and Xdh were also up-regulated in both models further demonstrating Hepa1c1c7 cells as a suitable model for investigating TCDD-regulated induction of xenobiotic metabolizing genes. In addition to these genes, the responses of Nqo1, Ugdh and Tnfaip2 were also conserved across models and were categorized as class I genes (similarly induced in both models; Figure 4B and 4C). However, Gsta2 was induced *in vivo *while no significant effect was detected in Hepa1c1c7 cells, and Ugt1a2 was induced *in vitro *but not differentially expressed in C57BL/6 hepatic tissue. Although many phase I and II metabolizing enzyme responses were conserved, differences exist that may limit Hepa1c1c7 cells from accurately modeling the full spectrum of *in vivo *hepatic responses elicited by TCDD.

A direct comparison of untreated Hepa1c1c7 cells and C57BL/6 hepatic tissue was performed to further investigate innate differences between the two models. Comparison of the normalized signal intensities revealed a good correlation (R = 0.75) between *in vitro *and *in vivo *basal expression levels (Figure 5). This illustrates that many genes are basally expressed to similar levels in both models as illustrated by the cluster of class I (similarly induced genes) closely surrounding the fitted line. Although a correlation exists, there are still differences in basal expression which may be associated with the origins of the models (i.e. normal hepatic tissue versus hepatoma derived Hepa1c1c7 cells), as well as the inability of *in vitro *systems to effectively model complex interactions between different cell types (e.g. Kupffer and stellate cells). For example, Myc, a G_1 _to S phase cell cycle regulator [23,26-29], was repressed *in vitro *while being induced *in vivo *and the model-specific responses may be related to difference in basal expression levels between the two models (Table 1). The levels of Myc transcripts in untreated Hepa1c1c7 cells were higher relative to untreated C57BL/6 hepatic tissue, consistent with the proliferative state of the *in vitro *system (data not shown). Examination of other class III genes suggests that they are more highly expressed *in vitro *when compared to *in vivo *(Figure 5). Consequently, differences in basal expression may be a factor contributing to divergent *in vitro – in vivo *responses. Another possible source for the model-specific responses may be related to DNA methylation status of the promoter region of TCDD-responsive genes in either model. DNA methylation results in gene silencing [40,41] and a previous study with Hepa1c1c7 has shown that TCDD-elicited gene expression responses are influenced by DNA methylation status [42]. The differing methylation states between the *in vitro *and *in vivo *systems may further contribute to the model-specific gene expression responses.

Many *in vitro *specific gene expression responses elicited by TCDD were associated with cell cycle progression and cell cycle arrest. Myc and its downstream target, cyclin D1, which forms a kinase complex with Cdk4 [43,44] were both repressed by TCDD. In contrast, Cdkn1a, an inhibitor of cyclin-dependent kinase 2 (Cdk2)-cyclin E complex kinase activity [43], was induced. Inactivation of the Cdk2-cyclin E complex prevents the phosphorylation of pRb resulting in cell cycle arrest during G_1_. Additionally, the *in vitro *induction of Btg2 suggests an alternative mechanism for cell cycle arrest during the G_2 _phase. Constitutively active BTG2 in human leukemia U937 cells, induces G_2_/M cell cycle arrest by inhibiting the formation of the cyclin B1 and Cdc2 complex, thereby inhibiting the active kinase function of the complex [30]. Collectively, these results corroborate and extend previous *in vitro *TCDD-mediated cell cycle arrest studies [45-48].

TCDD treatment resulted in a number of divergent gene responses across both models as represented by classes III and IV (Figures 4B and 4C). Genes related to immune cell accumulation, including major histocompatibility complex (MHC) molecules were only observed *in vivo*, and are likely a response to hepatic damage mediated by ROS or fatty accumulation and therefore independent of direct AhR action [19]. This is characteristic of the complex interaction between different cell types responding to liver injury that cannot be modeled in homogenous cultures of cells.

Pharmacokinetics may also contribute to response differences between the two models. Hepa1c1c7 cells were directly treated, whereas *in vivo*, TCDD must first be delivered to the liver and targeted cells prior to eliciting its effects. Additionally, C57BL/6 studies were able to be carried out to 168 hrs following TCDD treatment, while *in vitro *studies were limited to 48 hrs to minimize potentially confounding effects due to cell confluency. However, early responses associated with classes I and II (induced or repressed in both models; Figure 4B and 4C) are well conserved and exhibit comparable levels of induction or repression in both models. Hierarchical clustering of the common active genes (Figure 4C) illustrates gene induction occurs early while gene repression occurs later in both models. Clustering across both time and model revealed that gene expression profiles at 1 hr *in vitro *and 2 hr *in vivo *were most similar. This clustering pattern implies that early *in vitro *responses may accurately model early *in vivo *gene expression effects.

## Conclusion

Comparative analysis of global gene expression from Hepa1c1c7 cells and hepatic tissue from C57BL/6 mice identified several model-specific responses to TCDD that should be considered when extrapolating *in vitro *results to potential *in vivo *effects. Despite these differences, immortalized cells as well as other emerging *in vitro *systems (e.g., primary cells, stem cells and 3-D culture systems) provide valuable mechanistic information that supports the further development of high-throughput toxicity screening assays. However, the relevance of *in vitro *responses requires complementary *in vivo *verification. Furthermore, comparative studies exploiting other *in vitro *and *in vivo *systems, different structurally diverse ligands and other relevant model species will not only corroborate the relevance of the mechanisms, but will also support more appropriate extrapolations between rodent studies and potential effects in humans and ecologically-relevant species.

## Methods

### Culture and treatment of cell lines

Hepa1c1c7 wild-type and c4 ARNT-deficient cell lines (gifts from O. Hankinson, University of California, Los Angeles, CA) were maintained in phenol-red free DMEM/F12 media (Invitrogen, Carlsbad, CA) supplemented with 5% fetal bovine serum (FBS) (Hyclone, Logan, UT), 2.5 μg/mL amphotericin B (Invitrogen), 2.5 μg/mL amphotericin B (Invitrogen), 50 μg/mL gentamycin (Invitrogen), 100 U/mL penicillin and 100 μg/mL streptomycin (Invitrogen). 1 × 10^6^ cells were seeded into T175 culture flasks (Sarstedt, Newton, NC) and incubated under standard conditions (5% CO_2_, 37°C). Time course studies were performed with wild-type and c4 mutant cells where both were dosed with either 10 nM TCDD (provided by S. Safe, Texas A&M University, College Station, TX) or DMSO (Sigma, St. Louis, MO) vehicle and harvested at 1, 2, 4, 8, 12, 24 or 48 hrs. Additional untreated control cells were harvested at the time of dosing (i.e. 0 hrs). For the dose-response study, wild-type cells were treated with DMSO vehicle or 0.001, 0.01, 0.1, 1.0, 10 or 100 nM TCDD and harvested at 12 hrs. The treatment and harvesting regimen for cell culture studies are illustrated in Additional file 7.

### Animal treatment

The handling and treatment of female C57BL/6 mice has been previously described [19]. Briefly, immature ovariectomized mice were orally gavaged with 30 μg/kg TCDD for the time course study and sacrificed at 2, 4, 8, 12, 18, 24 72 or 168 hrs after treatment. For the dose-response study, mice were treated with 0.001, 0.01, 0.1, 1, 10, 100 or 300 μg/kg TCDD and sacrificed 24 hrs after dosing. Animals were sacrificed by cervical dislocation and tissue samples were removed, weighed, flash frozen in liquid nitrogen and stored at -80°C until further use.

### RNA isolation

Cells were harvested by scraping in 2.0 mL of Trizol Reagent (Invitrogen). Frozen liver samples (approximately 70 mg) were transferred to 1.0 mL of Trizol Reagent and homogenized in a Mixer Mill 300 tissue homogenizer (Retsch, Germany). Total RNA from each study was isolated according to the manufacturer's protocol with an additional acid phenol:chloroform extraction. Isolated RNA was resuspended in The RNA Storage Solution (Ambion Inc., Austin, TX), quantified (A_260_), and assessed for purity by determining the A_260_/A_280 _ratio and by visual inspection of 1.0 μg on a denaturing gel.

### Microarray experimental design

Changes in gene expression were assessed using customized cDNA microarrays containing 13,362 features representing 8,284 unique genes. For the time course study, TCDD-treated samples were compared to time-matched vehicle controls using an independent reference design [49]. In this design, treated Hepa1c1c7 cell or hepatic tissue samples were compared to the corresponding time-matched vehicle control with two independent labelings (dye swaps; Additional file 8). Four replicates of this design were performed, each using independent cell culture samples or different animals. Dose-response changes in gene expression were analyzed using a common reference design in which samples from TCDD-treated cells or mice were co-hybridized with a common vehicle reference (i.e. independent DMSO treated Hepa1c1c7 cell samples, hepatic samples from independent sesame oil treated C57BL/6 mice) using two independent labelings (Additional file 8). Four replicates with two independent labelings were performed for both *in vitro *and *in vivo *samples. Co-hybridizations of untreated Hepa1c1c7 cells and hepatic tissue from C57BL/6 mice were performed to investigate differences in basal gene expression levels between models (Additional file 8). Four replicates were performed with two independent labelings per sample (dye swap).

More detailed protocols regarding the microarray assay, including microarray preparation, labeling of the cDNA probe, sample hybridization and washing can be obtained from the dbZach website [50]. Briefly, polymerase chain reaction (PCR) amplified cDNAs were robotically arrayed onto epoxy-coated glass slides (Schott-Nexterion, Duryea, PA) using an Omnigrid arrayer (GeneMachines, San Carlos, CA) equipped with 48 (4 × 12) Chipmaker 2 pins (Telechem) at Michigan State University's Research Technology Support Facility [51]. Total RNA (30 μg) was reverse transcribed in the presence of Cy3- or Cy5-deoxyuridine triphosphate (dUTP) to create fluorescence-labeled cDNA, which was purified using a Qiagen PCR kit (Qiagen, Valencia, CA). Cy3 and Cy5 samples were mixed, vacuum dried and resuspended in 48 μL of hybridization buffer (40% formamide, 4× SSC, 1% sodium dodecyl sulfate [SDS]) with 20 μg polydA and 20 μg of mouse COT-1 DNA (Invitrogen) as competitor. This probe mixture was heated at 95°C for 3 min and hybridized on the array under a 22 × 60 mm LifterSlip (Erie Scientific Company, Portsmouth, NH) in a light-protected and humidified hybridization chamber (Corning Inc., Corning, NY) for 18–24 hrs in a 42°C water bath. Slides were then washed, dried by centrifugation and scanned at 635 nm (Cy5) and 532 nm (Cy3) on an Affymetrix 428 Array Scanner (Santa Clara, CA). Images were analyzed for feature and background intensities using GenePix Pro 5.0 (Molecular Devices, Union City, CA).

### Microarray data quality assurance, normalization and analysis

Microarray data were first passed through a quality assurance protocol prior to further analysis to ensure consistently high quality data throughout the dose-response and time course studies prior to normalization and further analysis [52]. All the collected data were then normalized using a semi-parametric approach [53]. Empirical Bayes analysis was used to calculate posterior probabilities (P1(*t*) value) of activity on a per gene and time point or dose group basis using the model-based *t*-value [54]. The data were filtered using a P1(*t*) cutoff of 0.9999 and ± 1.5 fold change to identify the most robust changes in gene expression and to obtain an initial subset of differentially regulated genes for further investigation and data interpretation. Subsequent analysis included agglomerative hierarchical and *k*-means clustering using the standard correlation distance metric implemented in GeneSpring 6.0 (Silicon Genetics, Redwood City, CA). Functional categorization of differentially regulated genes were mined and statistically analyzed from Gene Ontology [55] using GOMiner [56].

### Quantitative real-time PCR analysis

For each sample, 1.0 μg of total RNA was reverse transcribed by Superscript II using an anchored oligo-dT primer as described by the manufacturer (Invitrogen). The cDNA (1.0 μL) was used as a template in a 30 μL PCR reaction containing 0.1 μM of forward and reverse gene-specific primers designed using Primer 3 [57], 3 mM MgCl_2_, 1.0 mM dNTPs, 0.025 IU AmpliTaq Gold, and 1× SYBR Green PCR buffer (Applied Biosystems, Foster City, CA). PCR amplification was conducted in MicroAmp Optical 96-well reaction plates (Applied Biosystems) on an Applied Biosystems PRISM 7000 Sequence Detection System under the following conditions: initial denaturation and enzyme activation for 10 min at 95°C, followed by 40 cycles of 95°C for 15 s and 60°C for 1 min. A dissociation protocol was performed to assess the specificity of the primers and the uniformity of the PCR-generated products. Each plate contained duplicate standards of purified PCR products of known template concentration covering 7 orders of magnitude to interpolate relative template concentrations of the samples from the standard curves of log copy number versus threshold cycle (Ct). No template controls (NTC) were also included on each plate. Samples with a Ct value within 2 standard deviations of the mean Ct values for the NTCs were considered below the limits of detection. The copy number of each unknown sample for each gene was standardized to the geometric mean of three house-keeping genes (β-actin, Gapd and Hprt) to control for differences in RNA loading, quality, and cDNA synthesis. For graphing purposes, the relative expression levels were scaled such that the expression level of the time-matched control group was equal to 1. Statistical analysis was performed with SAS 8.02 (SAS Institute, Cary, NC). Data were analyzed by analysis of variance (ANOVA) followed by Tukey's *post hoc *test. Differences between treatment groups were considered significant when *p *< 0.05. Official gene names and symbols, RefSeq and Entrez Gene IDs, forward and reverse primer sequences, and amplicon sizes are listed in Table 4.

**Table 4:** Gene names and primer sequences for QRTPCR

RefSeq	Gene name	Gene Symbol	Entrez Gene ID	Forward Primer	Reverse Primer	Product Size (bp)
NM_007393	actin, beta, cytoplasmic	Actb	11461	GCTACAGCTTCACCACCACA	TCTCCAGGGAGGAAGAGGAT	123
NM_009992	cytochrome P450, family 1, subfamily a, polypeptide 1	Cyp1a1	13076	AAGTGCAGATGCGGTCTTCT	AAAGTAGGAGGCAGGCACAA	140
NM_010634	fatty acid binding protein 5, epidermal	Fabp5	16592	TGTCATGAACAATGCCACCT	CTGGCAGCTAACTCCTGTCC	87
NM_008084	glyceraldehyde-3- phosphate dehydrogenase	Gapd	2597	GTGGACCTCATGGCCTACAT	TGTGAGGGAGATGCTCAGTG	125
NM_013556	hypoxanthine phosphoribosyl transferase	Hprt	24465	AAGCCTAAGATGAGCGCAAG	TTACTAGGCAGATGGCCACA	104
NM_010849	myelocytomatosis oncogene	Myc	17869	CTGTGGAGAAGAGGCAAACC	TTGTGCTGGTGAGTGGAGAC	127
NM_011723	xanthine dehydrogenase	Xdh	22436	GTCGAGGAGATCGAGAATGC	GGTTGTTTCCACTTCCTCCA	124

## Authors' contributions

*In vitro *work and associated microarrays and QRTPCR were conducted by ED, similarly, *in vivo *studies and associated microarrays and QRTPCR were performed by DRB. LDB provided the normalization, statistical analysis and database support of microarray data. Comparison of *in vitro *and *in vivo *data was primarily carried out by ED with support by DRB. ED produced the initial draft of the manuscript. TRZ was responsible for the design and oversaw the completion of the study.

## Supplementary Material

Additional File 1 **Hepalclc7 TCDD time course microarray data**. Ratios represent expression relative to the time matched vehicle control. P1(*t*)-values represent posterior probabilities of activity on a per gene and time-point basis using the model-based t-value. Click here for file

Additional File 2 **Hepalclc7 TCDD dose-response microarray data**. Ratios represent expression relative to the time matched vehicle control. P1(*t*)-values represent posterior probabilities of activity on a per gene and dose basis using the model-based t-value. Click here for file

Additional File 3 **C57BL/6 mice hepatic tissue TCDD time course microarray data**. Ratios represent expression relative to the time matched vehicle control. P1(*t*)-values represent posterior probabilities of activity on a per gene and time-point basis using the model-based t-value. Click here for file

Additional File 4 **C57BL/6 mice hepatic tissue TCDD dose-response microarray data**. Ratios represent expression relative to the time matched vehicle control. P1(*t*)-values represent posterior probabilities of activity on a per gene and dose basis using the model-based t-value. Click here for file

Additional File 5 **Untreated Hepalclc7 and C57BL/6 sample microarray data**. Ratios represent basal expression of Hepalclc7 cells relative to hepatic tissue from C5VBL/6 mice. P1(*t*)-values represent posterior probabilities of activity on a per gene and dose basis using the model-based t-value. Click here for file

Additional File 6 **Gene names and primer sequences (5'-3') for transcripts verified by QRTPCR**. Primer pair sequences used to verify *in vitro *and *in vivo *microarray results using QRTPCR.
Click here for file

Additional File 7 **Hepa1c1c7 TCDD treatment and harvesting regimen**. For the time course study, wild-type and ARNT-deficient c4 mutant cells were treated with 10 nM TCDD or 0.1% DMSO vehicle and harvested at 1, 2, 4, 8, 12, 24, or 48 hrs post-treatment. Untreated controls were harvested at 0 hrs (as indicated by *). The dose-response study was done performed with Hepa1c1c7 wild-type cells and treated with 0.001, 0.01, 0.1, 1.0, 10, 100 nM TCDD or 0.1% DMSO vehicle and harvested 12 hrs post-treatment (as indicated by ‡). Click here for file

Additional File 8 **Microarray experimental designs for A) temporal, B) dose-response and C) basal expression studies**. A) Temporal gene expression patterns were analyzed by an independent reference design in which cells treated with TCDD (T) were co-hybridized to time-matched vehicle controls (V). This design involves two independent labelings per sample for a total of 14 arrays per replicate. Four biological replicates were conducted for a total of 56 microarrays. Numbers indicate time points for comparison. B) Dose-dependent changes in gene expression were analyzed 12 hrs after treatment using a common reference design in which cells treated with TCDD were co-hybridized with a common vehicle control. This design involves two independent labelings per sample for a total of 12 arrays per replicate. Four biological replicates were conducted for a total of 48 microarrays. Numbers indicate TCDD concentration in nM units. C) Comparative basal gene expression levels between untreated *in vitro *and *in vivo *samples were analyzed by an independent reference design. Four biological replicates of untreated Hepa1c1c7 cells and hepatic tissue from C57BL/6 mice harvested at 0 hrs were co-hybridized and two independent labelings were performed per sample for a total of 8 arrays. Double-headed arrows indicate dye swaps (each sample labeled with Cy3 and Cy5 on different microarrays). Click here for file
