# Supplementary figures and images for: In vivo – in vitro toxicogenomic comparison of TCDD-elicited gene expression in Hepa1c1c7 mouse hepatoma cells and C57BL/6 hepatic tissue
Source: BMC Genomics. 2006 Apr 12;7:80. doi: 10.1186/1471-2164-7-80 (PMC1513214; doi:10.1186/1471-2164-7-80)

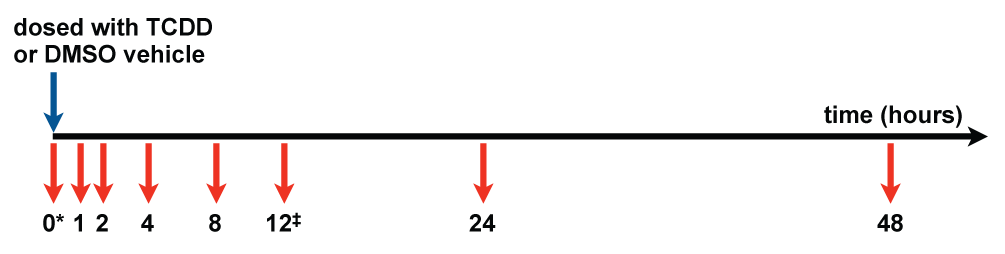

Supplement: Additional File 7 — Hepa1c1c7 TCDD treatment and harvesting regimen. For the time course study, wild-type and ARNT-deficient c4 mutant cells were treated with 10 nM TCDD or 0.1% DMSO vehicle and harvested at 1, 2, 4, 8, 12, 24, or 48 hrs post-treatment. Untreated controls were harvested at 0 hrs (as indicated by *). The dose-response study was done performed with Hepa1c1c7 wild-type cells and treated with 0.001, 0.01, 0.1, 1.0, 10, 100 nM TCDD or 0.1% DMSO vehicle and harvested 12 hrs post-treatment (as indicated by ‡). [file 1471-2164-7-80-S7.png]

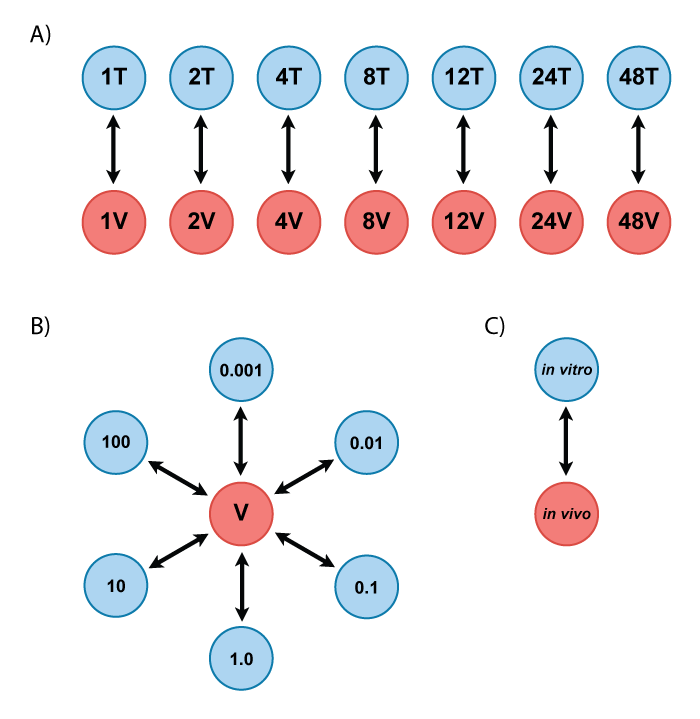

Supplement: Additional File 8 — Microarray experimental designs for A) temporal, B) dose-response and C) basal expression studies. A) Temporal gene expression patterns were analyzed by an independent reference design in which cells treated with TCDD (T) were co-hybridized to time-matched vehicle controls (V). This design involves two independent labelings per sample for a total of 14 arrays per replicate. Four biological replicates were conducted for a total of 56 microarrays. Numbers indicate time points for comparison. B) Dose-dependent changes in gene expression were analyzed 12 hrs after treatment using a common reference design in which cells treated with TCDD were co-hybridized with a common vehicle control. This design involves two independent labelings per sample for a total of 12 arrays per replicate. Four biological replicates were conducted for a total of 48 microarrays. Numbers indicate TCDD concentration in nM units. C) Comparative basal gene expression levels between untreated in vitro and in vivo samples were analyzed by an independent reference design. Four biological replicates of untreated Hepa1c1c7 cells and hepatic tissue from C57BL/6 mice harvested at 0 hrs were co-hybridized and two independent labelings were performed per sample for a total of 8 arrays. Double-headed arrows indicate dye swaps (each sample labeled with Cy3 and Cy5 on different microarrays). [file 1471-2164-7-80-S8.png]
